# Chikungunya outbreak in Bangladesh (2017): sociodemographic and clinical characteristics of patients from three hotspots

**DOI:** 10.1186/s41182-022-00399-3

**Published:** 2022-01-12

**Authors:** Mohammad Robed Amin, Mohammad Jahid Hasan, Md. Abdullah Saeed Khan, Md Abdur Rafi, Mohammad Rafiqul Islam, Tarek Shams, Mohammed Jahedul Islam, Abu Saif Mohammad Lutful Kabir, Mohiuddin Sharif, David Gozal

**Affiliations:** 1 Department of Medicine, Dhaka Medical College and Hospital-2, Room No 502, Dhaka, Bangladesh; 2 Pi Research Consultancy Center, Dhaka, Bangladesh; 3 Department of Medicine, Shaheed Suhrawardy Medical College, Dhaka, Bangladesh; 4 Department of Medicine, Cox’s Bazar Medical College, Cox’s Bazar, Bangladesh; 5 Mirsarai Upazilla Health Complex, Chattogram, Bangladesh; 6 Department of Medicine, Dhaka Medical College and Hospital-2, Dhaka, Bangladesh; 7 Department of Child Health, MU Women’s and Children’s Hospital University of Missouri School of Medicine, Columbia, MO USA

**Keywords:** Chikungunya, Clinical features, Outbreak, Hotspot, Tropical disease

## Abstract

**Background:**

Chikungunya is a severely debilitating disease. Bangladesh witnessed one of the largest outbreaks in 2017. Here, we described the clinical profile of the chikungunya outbreak in Bangladesh and its heterogeneity across three hotspots.

**Methods:**

This was a descriptive cross-sectional study of 432 individuals interviewed from the outpatient department of three study sites (Dhaka, Chittagong, and Sitakundu Upazilla of Bangladesh) after confirmation by the study physicians. Both laboratory-confirmed cases and probable cases were recruited between July and October 2017.

**Results:**

Of all, 18% (79) were laboratory confirmed, and 353 82% (335) were probable cases. The male:female ratio was almost equal (1.09:1), and the predominant age group was 18–59 years. The mean age of the presentation was 36.07 ± 13.62 (SD) years. Fever and arthralgia were the most common presentations and were present in > 95% of cases. Other frequent symptoms were fatigue, myalgia, headache, nausea, and vomiting. Approximately half of the patients had arthritis and erythematous rash. Arthritis was predominant in Chittagong city, while maculopapular rash was not observed in Sitakunda city. However, fatigue, nausea, and vomiting are more common among patients in Dhaka city. Significant heterogeneity of clinical manifestations was present across the three hotspots (*p* < 0.05 for all). Both confirmed and probable cases shared similar characteristics except muscle ache (*p* = 0.22) and rash (*p* = 0.37).

**Conclusion:**

The clinical profile of chikungunya virus-induced disease displays significant location-related heterogeneity in Bangladesh during a large outbreak. Although the causes of such differences are unclear, improved public and medical personnel education on this condition may lead to earlier diagnosis and treatment.

## Background

Chikungunya fever (CF) is caused by the chikungunya virus (CHIKV), which is transmitted by *Aedes* mosquitoes [1]. The disease was first reported in Tanzania, and the name is derived from the ‘Makonde dialect’, which means ‘that which bends up’, about the stooped posture adopted by patients as a result of the arthritic symptoms of the disease [1, 2]. Humans may serve as reservoirs of this virus during epidemics, while several vertebrates, including monkeys, rodents, birds, etc., act as reservoirs during interepidemic periods [3].

CF has been reported from approximately 60 countries from different parts of the globe [4, 5], with the most recent outbreak in 2017 in Bangladesh [6]. Historically, the first reported CF outbreak was observed in 2008 in two villages in the northern part of the country [7], with two subsequent small-scale outbreaks in rural communities in 2011 and 2012 [8, 9]. Dhaka, the capital of Bangladesh, experienced the most devastating and massive outbreak of CF during the period of April–September 2017, with documented > 13,000 clinically confirmed cases in the city of Dhaka. It also spreads into 17 other districts in the country, and an estimate suggested that it risks over 2 million people becoming infected by the virus [10]. Notably, another large port city, Chittagong, was also affected as an extension of the outbreak in September 2017, followed a month later by further extension of the outbreak to Upazilla Sitakundu in October 2017 [11, 12].

In general, CF is a self-limited disease in which the two most common features include fever and arthralgia/arthritis [13]. CF may also be accompanied by a rash, which can range in severity from a mild, localized rash to an extensive rash involving > 90% of the body surface [14]. Due to the multiplicity of CF symptoms, patients will mostly seek medical care for joint pain, swelling, and morning stiffness, which may persist for a prolonged period, often lasting even years [6, 14, 15]. A significant portion of patients also experience depression, sleeping disorders, and mood swings [15, 16]. In addition, a minority of CF patients will report chronic joint complaints [15].

The clinical manifestation of chikungunya fever is well documented in the literature [6, 9, 17, 18]. However, during the outbreak, it was observed that the clinical profile of the patients displayed location-related heterogeneity. To the best of our knowledge, the published reports on the 2017 chikungunya fever outbreak have not focused on the heterogeneity of clinical profiles during the acute phase of illness as a function of their area of residence. Therefore, delineating the clinical manifestation of the 2017 chikungunya outbreak and examining its heterogeneity across the hotspots were the objectives of the study.

## Materials and methods

### Setting and participants

**Fig. 1:**
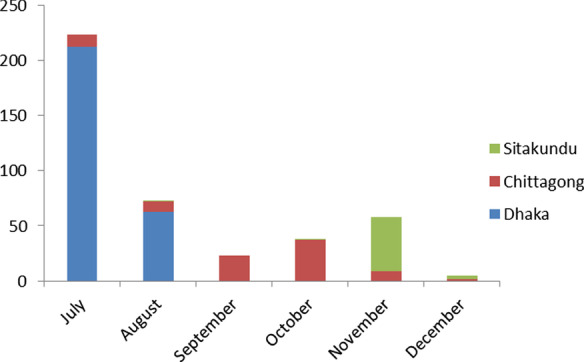
Time trend of patient inclusion from three hotspots

This cross-sectional study was conducted in the medicine outpatient of the three major hospitals of the hotspots: Dhaka Medical College Hospital (DMCH), Dhaka, Chittagong Medical College Hospital (CMCH), Chittagong and Sitakundu Upazilla Health Complex (SUHC), Sitakundu. Although a total of 17 districts of Bangladesh were affected by the outbreak, they were highly clustered in these three regions. In Dhaka, the chikungunya cases started to rise from the 1st week of May and continued until late August 2017 [19]. However, after all, administrative procedures, we started our data collection from the 1st week of July 2017 in the selected hospitals of Dhaka and Chittagong. Since the last week of August, the cases declined in Dhaka, and we decided to stop further data collection in DMCH and continue in CMCH. Subsequently, an outbreak was notified in Sitakundu, a rural subdistrict of Chittagong by the local health authority in the 1st week of November, and we included SUHC for data collection. The time trend of patient inclusion from different areas is shown in Fig. 1.

All the patients attending the medicine outpatient department during the study period were the study population. Convenient sampling according to the inclusion and exclusion criteria was used to recruit patients. The inclusion criteria were confirmed or probable cases of chikungunya according to the definition of the national chikungunya guideline of Bangladesh aged ≥ 12 years. Possible cases of chikungunya, aged < 12 years, and patients with chronic arthralgia due to other known etiologies were excluded.

### Case definition

A confirmed chikungunya case was defined as a patient meeting the laboratory criteria, irrespective of the clinical presentation; a probable case was defined as a patient meeting both the clinical and epidemiological criteria, and a possible case was defined as a patient meeting only clinical criteria [4]. The clinical criteria adopted for case definition were: (i) acute onset of fever > 38.5 °C and (ii) severe arthralgia/arthritis not explained by other medical conditions; epidemiological criteria were: (i) residing or having visited epidemic areas and (ii) having reported transmission within 15 days prior to the onset of symptoms and laboratory criteria was at least one of the following tests in the acute phase: (i) virus isolation by cell culture, (ii) presence of viral RNA by real Time RT-PCR (within 5 days of onset of illness), (iii) presence of viral-specific IgM antibody in a single serum sample collected within 5–28 days of onset fever, and (iv) fourfold rise of IgG antibody in samples collected at least 3 weeks apart (1st sample after 7 days). As cell culture and RT-PCR tests were not available in our study center, we adopted the presence of IgM anti-CHIKV antibody in serum samples detected by ELISA (CHIKjj Detect™ IgM ELISA Kit, InBios International, Inc., USA) to define laboratory-confirmed cases.

### Data collection

A semistructured case report form (CRF) was used to collect data. Patients’ sociodemographic information, self-reported comorbidities, and clinical symptoms were collected by a face-to-face interview during the consultation. Further physical examination was conducted by the attending physician, and relevant clinical signs were inserted into the CRF. Being a cross-sectional study and due to lack of follow-up of the patients, the inclusion of laboratory investigations was not possible.

### Statistical analyses

Being an observational study descriptive statistics was used to ascertain the clinical profile of CF infection. Continuous variables were reported as percentages and means along with standard deviations. Moreover, to describe the categorical variables, frequency and percentage were used. For comparison of sociodemographic and clinical presentations among patients from different hotspots, Chi-square test for categorical variables and independent *t* test for continuous variables were used as appropriate. A *p* value < 0.05 was considered statistically significant. STATA version 16 was used for statistical analyses.

## Results

### Sociodemographic characteristics

A total of 432 chikungunya cases were enrolled in the study. Among them, 79 (18%) were laboratory-confirmed, and 353 (82%) were probable cases. Of all, 286 (66%) cases were from Dhaka city, 93 (21.5%) cases were from Chittagong city and 53 (12.5%) cases were from Sitakundu, a subdistrict situated in Chittagong district. The mean (SD) age was 36 (13.6) years, with the majority being adults. Male:female ratio was almost equal. The proportion of pediatric and female cases was higher in Sitakundu than in other regions. The prevalence of comorbidities such as type 2 diabetes mellitus and hypertension was similar in different regions (Table 1).

**Table 1:** Socio-demographic characteristics of the chikungunya patients from different hotspots (*n* = 432)

Characteristics	Total, *n* (%)	Dhaka, *n* (%)	Chittagong, *n* (%)	Sitakundu, *n* (%)	*p* value
Age, mean (SD) (years)	36.0 (13.6)	36.2 (13.3)	39.2 (11.3)	29.9 (16.7)	< 0.001
Age group (years)					
12–17	39 (9.0)	23 (8.0)	3 (3.2)	13 (24.5)	0.001
18–59	368 (85.2)	245 (85.6)	86 (92.4)	37 (69.8)	
> 59	25 (5.8)	18 (6.3)	4 (4.3)	3 (5.6)	
Sex					
Male	224 (51.8)	154 (53.8)	54 (58.0)	16 (30.2)	0.003
Female	208 (48.2)	132 (46.1)	39 (41.9)	37 (69.8)	
Education					
Illiterate	10 (2.3)	0 (0.0)	0 (0.0)	10 (18.8)	< 0.001
Primary	52 (12.0)	14 (4.9)	7 (7.5)	31 (58.5)	
High school	65 (15.0)	42 (14.7)	19 (20.4)	4 (7.5)	
Intermediate	76 (17.6)	58 (20.3)	17 (18.3)	1 (1.9)	
Graduate	145 (33.5)	97 (33.9)	47 (50.5)	1 (1.9)	
Missing	80 (18.5)	75 (26.2)	3 (3.2)	6 (11.3)	
Occupation					
Service	102 (23.6)	57 (19.9)	44 (47.3)	1 (1.9)	< 0.001
Businessman	52 (12.0)	40 (14.0)	11 (11.8)	1 (1.9)	
Farmer	1 (0.2)	0 (0.0)	0 (0.0)	1 (1.9)	
House wife	161 (37.2)	105 (36.7)	26 (28.0)	30 (56.6)	
Student	60 (13.9)	46 (16.1)	7 (7.5)	7 (13.2)	
Others	18 (4.2)	5 (1.7)	2 (2.1)	11 (20.7)	
Missing	38 (8.8)	33 (11.5)	3 (3.2)	2 (3.7)	
Religion					
Buddist	4 (0.9)	4 (1.4)	0 (0.0)	0 (0.0)	< 0.001
Hindu	79 (18.3)	16 (5.6)	13 (14.0)	50 (94.3)	
Islam	336 (77.8)	257 (89.9)	78 (83.8)	1 (1.9)	
Missing	13 (3.0)	9 (3.1)	2 (2.1)	2 (3.7)	
Marital status					
Divorced	1 (0.2)	0 (0.0)	0 (0.0)	1 (1.9)	0.023
Married	320 (74.1)	208 (72.7)	77 (82.8)	35 (66.0)	
Unmarried	85 (19.7)	57 (19.9)	14 (15.0)	14 (26.4)	
Widow	1 (0.2)	0 (0.0)	0 (0.0)	1 (1.9)	
Missing	25 (5.8)	21 (7.3)	2 (2.1)	2 (3.7)	
Residence					
Rural	60 (13.9)	11 (3.8)	1 (1.1)	48 (90.5)	< 0.001
Urban	347 (80.3)	254 (88.8)	90 (96.7)	3 (5.6)	
Missing	25 (5.8)	21 (7.3)	2 (2.1)	2 (3.7)	
Comorbidity					
CVD	5 (1.2)	1 (0.3)	4 (4.3)	0 (0.0)	0.017
DM	23 (5.3)	18 (6.3)	4 (4.3)	1 (1.9)	0.414
HTN	29 (6.7)	21 (7.3)	5 (5.4)	3 (5.6)	0.877

### Clinical presentation

The classic characteristic symptoms of chikungunya were observed in this series, indicating that most of the probable cases in the setting of the ongoing outbreak were chikungunya cases (Table 2). Ninety-six percent presented with fever and arthralgia, while fatigue, myalgia, arthritis, and headache were observed in 53%, 50%, 49%, and 38% of cases, respectively. The erythematous rash was observed in 47% of cases. Other presenting symptoms were retro-orbital pain, cough, and gastrointestinal upset (abdominal pain, nausea, vomiting, and diarrhea). Patients from Chittagong presented less frequently than fever compared to the other two regions, while fatigue was less commonly observed among the patients from Sitakundu. Nonspecific symptoms, such as abdominal pain, nausea, vomiting, and diarrhea, were predominant in Dhaka city compared with the two other sites (Table 2).

**Table 2:** Clinical presentations of the chikungunya patients from different regions (*n* = 432)

Clinical presentation	Total, *n* (%)	Dhaka, *n* (%)	Chittagong, *n* (%)	Sitakundu, *n* (%)	*p* value
Fever	413 (95.6)	279 (97.5)	83 (89.2)	51 (96.2)	0.005
Joint pain (arthralgia)	415 (96.0)	276 (96.5)	93 (100.0)	46 (86.8)	< 0.001
Joint swelling or arthritis	213 (49.3)	117 (40.9)	82 (88.2)	14 (26.4)	0.001
Muscle ache	216 (50.0)	132 (46.1)	47 (50.5)	37 (69.8)	0.006
Fatigue	228 (52.7)	198 (69.2)	29 (31.2)	1 (1.9)	< 0.001
Headache	165 (38.2)	117 (40.9)	17 (18.3)	31 (58.5)	< 0.001
Eye ache	32 (7.4)	32 (11.2)	0 (0.0)	0 (0.0)	< 0.001
Rash	201 (46.5)	141 (49.3)	45 (48.4)	15 (28.3)	0.016
Nausea	132 (30.5)	124 (43.3)	7 (7.5)	1 (1.9)	< 0.001
Vomiting	130 (30.1)	122 (42.6)	7 (7.5)	1 (1.9)	< 0.001
Diarrhea	38 (8.8)	38 (13.3)	0 (0.0)	0 (0.0)	< 0.001
Chills	14 (3.2)	14 (4.9)	0 (0.0)	0 (0.0)	0.018
Cough	48 (11.1)	46 (16.1)	2 (2.1)	0 (0.0)	< 0.001
Abdominal pain	58 (13.4)	58 (20.3)	0 (0.0)	0 (0.0)	< 0.001

Some differences were observed between the clinical features of laboratory-confirmed and probable cases of chikungunya. Fever was more commonly observed among the probable cases, while joint symptoms such as joint pain and swelling were more common in laboratory-confirmed cases. Nonspecific presentations, e.g., retroorbital pain, headache, and gastrointestinal symptoms, were more frequent in probable cases (Table 3).

**Table 3:** Clinical presentations of the chikungunya patients according to diagnosis (*n* = 432)

Clinical presentation	Confirmed cases, *n* (%)	Probable cases, *n* (%)	*p* value
Fever	69 (87.3)	344 (97.4)	0.001
Joint pain	79 (100.0)	336 (95.2)	0.030
Joint swelling	68 (86.1)	145 (41.1)	< 0.001
Muscle ache	36 (45.6)	180 (51.0)	0.228
Fatigue	24 (30.4)	204 (57.8)	< 0.001
Headache	12 (15.2)	153 (43.3)	< 0.001
Eye ache	0 (0.0)	32 (9.1)	0.001
Rash	35 (44.3)	166 (47.0)	0.378
Nausea	5 (6.3)	127 (36.0)	< 0.001
Vomiting	5 (6.3)	125 (35.4)	< 0.001
Diarrhea	0 (0.0)	38 (10.7)	< 0.001
Chills	0 (0.0)	14 (3.9)	0.056
Cough	2 (2.5)	46 (13.0)	0.003
Abdominal pain	0 (0.0)	58 (16.4)	< 0.001

The clinical characteristics of chikungunya cases showed a similar distribution according to age and sex, except joint swelling, which was less frequent among children, and less frequent erythematous rash among male patients (Table 4).

**Table 4:** Clinical presentations of the chikungunya patients according to age and sex (*n* = 432)

Clinical presentation	Age				Sex		
	< 18	18–59	> 59	*p* value	Male	Female	*p* value
Fever	38 (97.4)	350 (95.1)	25 (100.0)	0.770	212 (94.6)	201 (96.6)	0.313
Joint pain	35 (89.7)	355 (96.5)	25 (100.0)	0.102	212 (94.6)	203 (97.6)	0.115
Joint swelling	10 (25.6)	191 (51.9)	12 (48.0)	0.007	101 (45.1)	112 (53.8)	0.069
Muscle ache	24 (61.5)	180 (48.9)	12 (48.0)	0.322	106 (47.3)	110 (52.9)	0.248
Fatigue	18 (46.1)	197 (53.5)	13 (52.0)	0.675	115 (51.3)	113 (54.3)	0.534
Headache	18 (46.1)	141 (38.3)	6 (24.0)	0.205	81 (36.2)	84 (40.4)	0.367
Eye ache	2 (5.1)	29 (7.9)	1 (4.0)	0.787	17 (7.6)	15 (7.2)	0.881
Rash	13 (33.3)	181 (49.2)	7 (28.0)	0.026	93 (41.5)	108 (51.9)	0.030
Nausea	12 (30.7)	113 (30.7)	7 (28.0)	0.960	65 (29.0)	67 (32.2)	0.472
Vomiting	12 (30.7)	111 (30.2)	7 (28.0)	0.970	64 (28.5)	66 (31.7)	0.474
Diarrhea	4 (10.2)	32 (8.7)	2 (8.0)	0.938	23 (10.3)	15 (7.2)	0.262
Chills	0 (0.0)	13 (3.5)	1 (4.0)	0.489	5 (2.2)	9 (4.3)	0.219
Cough	4 (10.2)	42 (11.4)	2 (8.0)	0.857	32 (14.3)	16 (7.7)	0.029
Abdominal pain	6 (15.4)	48 (13.0)	4 (16.0)	0.853	30 (13.4)	28 (13.5)	0.983

## Discussion

The chikungunya outbreak that occurred in Bangladesh during 2017 was the largest outbreak since the first outbreak in 2008 [7]. Although the majority of the cases during this epidemic were reported from the Dhaka metropolitan area during May to late August [6, 9], the cases increased in another of the largest metropolitan cities, Chittagong, in subsequent months [20], and a clustered increase in cases was notified from a rural subdistrict, Sitakundu, during November [12]. We included patients from all these hotspots to describe the comprehensive characteristics of the epidemic patients. Our strategy could be justified by a recent investigation into the epidemic that reported that these three areas had the highest patient burden during the epidemic [19]. Although localized outbreaks affecting a cluster of patients were previously observed in the Dohar and Shibganj subdistricts in 2011 [8, 21], the repeated occurrence as the outbreak abated in the epicenter indicates the need for explorative epidemiological research that includes vector surveillance aiming to identify hidden pockets of transmission in rural areas.

Our study included 432 cases of chikungunya, including 79 confirmed and 353 probable cases based on clinical and epidemiological criteria. Although the clinical features of CF and dengue fever overlap [15, 22], the chikungunya outbreak was already ongoing and recognized by the time data collection was initiated, and no dengue fever cases were reported by the health authority during this period, which ensures that the current cohort exclusively consisted only of CF cases.

The age and sex distribution are remarkably similar to those reported in previous outbreaks [8, 21]. However, in the Sitakundu subdistrict, the proportion of females was significantly higher, likely reflecting occupational differences and the location of transmission [6]. Indeed, in a previous outbreak that occurred in 2012 in a village, women were 1.5 times more likely to be affected than men [23]. Children were less affected than adults in both rural and urban areas. Even in metropolitan areas (Dhaka and Chittagong), only a very small minority of cases were children. This phenomenon was explored by Salje et al., who noted that people living within or around their homes in a village setting (defined as within a 50 m radius) were more likely to be affected by chikungunya and that children were likely to be infected in the village because of their proximity to their mothers [23].

A comparative analysis of clinical presentations showed that confirmed cases less frequently presented with fever compared to probable cases. As an observational study, patients were included from the regular healthcare center visit. In addition, during the epidemic, clinical diagnosis was preferred for prompt management and reducing healthcare burden; most likely, patients with typical presentations, such as fever and arthralgia, were not sent for a confirmatory diagnostic test, which can result in such findings. This finding should be interpreted cautiously, as the symptoms of F and dengue may overlap among patients and the lack of confirmatory diagnosis could not roll out the probability of dengue or dengue-CF mixed infection. However, the WHO guidelines for Chikungunya management suggest that during an established epidemic, all patients need not be subjected to confirmatory tests. An epidemiologic link combined with typical clinical manifestation might be enough as clinical management does not differ between a probable case and a confirmed case [4]. According to this guideline, being a resource-poor setting, the patients presenting with typical symptoms were not confirmed through laboratory diagnosis, which might explain the difference in presentation between probable cases and confirmed cases, where probable cases were more likely to have typical presentations. Another study conducted in metropolitan Dhaka during the epidemic reported similar clinical presentations among confirmed and probable cases [6]. Clinical presentations of chikungunya were somewhat similar among different age and sex groups of the patients, consistent with the findings from other studies [6, 10, 24].

In all three outbreak epicenters of Bangladesh, fever and arthralgia were observed as the predominant symptoms (more than 90%), while during the examination, the predominant findings were arthritis and generalized erythematous rash, similar to other reports around the world [6, 8, 10, 20, 21]. However, nonspecific symptoms such as nausea, vomiting, abdominal discomfort, cough, and headache were reported predominantly in the Dhaka outbreak rather than in the Chittagong and Sitakundu outbreaks. The limited patient number from these centers could explain the findings, and diversity in the clinical presentation may be observed if the number of patients is increased. However, one possibility accounting for the variance in the clinical presentation may reflect temporal changes in the virus as the outbreak is prolonged and extends to different geographical locations. The erythematous generalized rash was predominantly seen in early febrile periods and is also found in other viral infections, such as dengue fever [25, 26]. Therefore, the rash may not provide a reliable sign for diagnosing CF during outbreaks. Arthritis was reported in approximately 50% of cases, irrespective of age and sex, but the severity and disabling morbidity of joint involvement especially joint swelling were preferentially present in older age.

Sustained fever was noted in all locations, and arthritis affected lower limb joints more often than upper limbs and was either oligoarticular (2–4 joints) or polyarticular (more than 5 joints), with monoarticular involvement not being identified during the acute phase of illness [6]. Arthralgia occurred in more than 90% of cases, and it is possible that arthritis was not present due to early introduction by patients of anti-inflammatory therapies that may have reduced joint swelling before seeking physician consultation.

## Limitations

Although our study provides an overview of the demographic and clinical profile of the patients of three different hotspots of the chikungunya outbreak of 2017 that occurred in Bangladesh, it has several limitations. Ideally, serological or RT-PCR confirmation would have been desirable. However, RT-PCR-based diagnosis was only possible at the Institute of Epidemiology, Disease Control and Research (IEDCR) in Dhaka, which was not readily accessible. More importantly, the study was conducted during the peak of the acute phase of the outbreak, therefore, justifying the inclusion of patients with classic symptoms and signs. In addition, describing the clinical features of a small number of case patients selected by convenience sampling may not provide the comprehensive characteristics of all affected cases during this epidemic and could not be generalized. Long-term follow-up of the cohort and specific investigation of the vectors and virus bionomics were not pursued and may shed some light on changes in the virus over time that may account for the heterogeneity of the clinical manifestations over time.

## Conclusions

The clinical features of chikungunya infection during the outbreak in Bangladesh in 2017 showed substantial heterogeneity in three hotspots. High-grade prolonged fever with oligo- or poly-articular arthralgia and arthritis along with generalized rash were the predominant clinical features of the disease, while nausea, vomiting, myalgia, diarrhea, and cough were more commonly seen in Dhaka than in the other outbreak locations.

## Data Availability

Available from the corresponding author, upon reasonable request.

## Abbreviations

CF: Chikungunya fever
CRF: Case Record Form
HSC: Higher Secondary Certificate
SSC: Secondary School Certificate

## References

[1] Chhabra M, Mittal V, Bhattacharya D, Rana U, Lal S (2008). Chikungunya fever: a re-emerging viral infection. Indian J Med Microbiol.

[2] Alphaviruses. Fields virology. Philadelphia: Lippincott-Raven; 1996.

[3] Chikungunya Fever Fact Sheet—CDC Division of Vector Borne Infectious Diseases.

[4] Guidelines on clinical management of chikungunya fever. https://www.who.int/publications/i/item/guidelines-on-clinical-management-of-chikungunya-fever. Accessed 5 Dec 2021.

[5] World Health Organization RO for S-EA (2008). Guidelines on clinical management of chikungunya fever.

[6] Hossain MS, Hasan MM, Islam MS, Islam S, Mozaffor M, Khan MAS (2018). Chikungunya outbreak (2017) in Bangladesh: clinical profile, economic impact and quality of life during the acute phase of the disease. PLoS Negl Trop Dis.

[7] ICDDR’B (2009). First identified outbreak of Chikungunya in Bangladesh, 2008. HealTH Sci Bull.

[8] Khatun S, Chakraborty A, Rahman M, Nasreen Banu N, Rahman MM, Hasan SMM (2015). An outbreak of chikungunya in rural Bangladesh, 2011. PLoS Negl Trop Dis.

[9] Kabir I, Dhimal M, Müller R, Banik S, Haque U (2017). The 2017 Dhaka chikungunya outbreak. Lancet Infect Dis.

[10] Anwar S, Mourosi JT, Khan MF, Ullah MO, Vanakker OM, Hosen MJ (2020). Chikungunya outbreak in Bangladesh (2017): clinical and hematological findings. PLoS Negl Trop Dis.

[11] Anwar S, Mourosi JT, Khan MF, Ullah MO, Vanakker OM, Hosen MJ (2017). Chikungunya outbreak in Bangladesh (2017): clinical and hematological findings. PLoS Negl Trop Dis.

[12] Hussain A. Chikungunya strikes fishing community in Chittagong’s Sitakunda. Dhaka Tribune. 2017.

[13] Rahim MA, Uddin KN (2017). Chikungunya: an emerging viral infection with varied clinical presentations in Bangladesh: reports of seven cases. BMC Res Notes.

[14] Miner JJ, Aw Yeang HX, Fox JM, Taffner S, Malkova ON, Oh ST (2015). Brief Report: Chikungunya viral arthritis in the United States: a mimic of seronegative rheumatoid arthritis. Arthritis Rheumatol.

[15] Amin MR, Rahman MM, Islam QT (2017). Chikungunya. J Med.

[16] Schilte C, Staikovsky F, Couderc T, Madec Y, Carpentier F, Kassab S (2013). Chikungunya virus-associated long-term arthralgia: a 36-month prospective longitudinal study. PLoS Negl Trop Dis.

[17] Kabir R, Rahman S, Kalim T, Arafat S, Monte-Serrat D (2017). Chikungunya fever: an emerging public health problem in Bangladesh. J Sci Res Rep.

[18] Rabbani SB, Saha PR, Hossain MI, Begum A, Talukder MJ (2019). A clinical study on chikungunya fever in a multidisciplinary hospital of Dhaka City. J Bangladesh Coll Physicians Surg.

[19] Mahmud AS, Kabir MI, Engø-Monsen K, Tahmina S, Riaz BK, Hossain MA (2021). Megacities as drivers of national outbreaks: the 2017 chikungunya outbreak in Dhaka Bangladesh. PLoS Negl Trop Dis.

[20] Biswas B, Amin S, Azad MAK, Billah M, Murad M, Chowdhury S (2019). A study on the dangerous outbreak of chikungunya in Chittagong, including a limited survey around that city of Bangladesh. Int J Community Med Public Health.

[21] Haque F, Rahman M, Banu NN, Sharif AR, Jubayer S, Shamsuzzaman A (2019). An epidemic of chikungunya in northwestern Bangladesh in 2011. PLoS ONE.

[22] Guzmán MG, Kouri G (2002). Dengue: an update. Lancet Infect Dis.

[23] Salje H, Lessler J, Paul KK, Azman AS, Rahman MW, Rahman M (2016). How social structures, space, and behaviors shape the spread of infectious diseases using chikungunya as a case study. Proc Natl Acad Sci USA.

[24] Lakshmi V, Neeraja M, Subbalaxmi MVS, Parida MM, Dash PK, Santhosh SR (2008). Clinical features and molecular diagnosis of chikungunya fever from South India. Clin Infect Dis.

[25] Hasan MJ, Tabassum T, Sharif M, Khan MAS, Bipasha AR, Basher A (2021). Clinico-epidemiologic characteristics of the 2019 dengue outbreak in Bangladesh. Trans R Soc Trop Med Hyg.

[26] Rafi A, Mousumi AN, Ahmed R, Chowdhury RH, Wadood A, Hossain G (2020). Dengue epidemic in a non-endemic zone of Bangladesh: clinical and laboratory profiles of patients. PLoS Negl Trop Dis.

