# Correction to: Chikungunya outbreak in Bangladesh (2017): sociodemographic and clinical characteristics of patients from three hotspots

**DOI:** 10.1186/s41182-022-00403-w

**Published:** 2022-01-24

**Authors:** Mohammad Robed Amin, Mohammad Jahid Hasan, Md. Abdullah Saeed Khan, Md Abdur Raf, Mohammad Rafiqul Islam, Tarek Shams, Mohammed Jahedul Islam, Abu Saif Mohammad Lutful Kabir, Mohiuddin Sharif, David Gozal

**Affiliations:** 1 Department of Medicine, Dhaka Medical College and Hospital-2, Room No 502, Dhaka, Bangladesh; 2 Pi Research Consultancy Center, Dhaka, Bangladesh; 3 Department of Medicine, Shaheed Suhrawardy Medical College, Dhaka, Bangladesh; 4 Department of Medicine, Cox’s Bazar Medical College, Cox’s Bazar, Bangladesh; 5 Mirsarai Upazilla Health Complex, Chattogram, Bangladesh; 6 Department of Medicine, Dhaka Medical College and Hospital-2, Dhaka, Bangladesh; 7 Department of Child Health, MU Women’s and Children’s Hospital University of Missouri School of Medicine, Columbia, MO USA

## Correction to: Tropical Medicine and Health (2022) 50:9 10.1186/s41182-022-00399-3

Following publication of the original article [1], the authors identified an error in the author name of Mohammad Rafiqul Islam.

The incorrect author name is: Rafiqul Islam.

The correct author name is: Mohammad Rafiqul Islam.

The author group has been updated above and the original article [1] has been corrected.
